# Safety of vacuum-assisted biopsy/mammatome guided, non-operative management of B3 lesions with atypia: a 7-year follow-up study

**DOI:** 10.1186/bcr3793

**Published:** 2015-11-05

**Authors:** Alex Wilkins, Peter Kneeshaw, Penelope McManus, Kartikae Grover, Caroline Bradley, Ayesha Rahman, Anne Hubbard

**Affiliations:** 1 Castle Hill Hospital, Cottingham, UK; 2 Hull and East Yorkshire Medical School, Hull, UK

## Introduction

B3 management balances safe treatment of potential malignancy against the morbidity of surgical excision of benign lesions. Vacuum-assisted biopsy (VAB) increases diagnostic accuracy, removing some lesions entirely without surgery. Few follow-up data are available to assess the safety and effectiveness of this approach.

## Methods

A total of 129 patients with B3 VAB with atypia were identified using Labcentre histopathology codes at a single centre. Hospital and NBSS records were analysed to identify patients treated with VAB and mammographic surveillance alone and to determine outcome over a follow-up period of 52−142 months (median 85).

## Results

Ten per cent progressed directly to surgery (13/129). A total of 116 were followed mammographically after VAB with no surgical intervention (49 ADH, 2 ALH, 21 LCIS, 44 atypia (not otherwise specified)). Nine patients re-presented to the service with invasive carcinoma (six ipsilateral) and two with DCIS (both ipsilateral) between 12 and 80 months. The ipsilateral re-presentation rate was highest for ADH (5/49) and LCIS (2/21). In the absence of ADH or LCIS, the only ipsilateral representation was one low-grade DCIS, 62 months after VAB.

## Conclusion

Re-presentation with ipsilateral carcinoma following VAB excision for ADH and LCIS is comparable to surgical excision for ADH and LCIS. National guidance is required.

